# Astaxanthin Activates Nuclear Factor Erythroid-Related Factor 2 and the Antioxidant Responsive Element (Nrf2-ARE) Pathway in the Brain after Subarachnoid Hemorrhage in Rats and Attenuates Early Brain Injury

**DOI:** 10.3390/md12126125

**Published:** 2014-12-18

**Authors:** Qi Wu, Xiang-Sheng Zhang, Han-Dong Wang, Xin Zhang, Qing Yu, Wei Li, Meng-Liang Zhou, Xiao-Liang Wang

**Affiliations:** 1 Department of Neurosurgery, Jinling Hospital, School of Medicine, Nanjing University, Nanjing 210002, China; E-Mails: 18652021010@163.com (Q.W.); zhangxssp@163.com (X.-S.Z.); lwxzlw@gmail.com (W.L.); mlzhou1979@hotmail.com (M.-L.Z.); wangxiaoliang1234@hotmail.com (X.-L.W.); 2 Department of Clinical Laboratory, Affiliated Hospital of Nanjing University of Chinese Medicine, Nanjing 210029, China; E-Mail: njyuqing@me.com

**Keywords:** astaxanthin, early brain injury, subarachnoid hemorrhage, nuclear factor erythroid-related factor 2

## Abstract

Astaxanthin (ATX) has been proven to ameliorate early brain injury (EBI) after experimental subarachnoid hemorrhage (SAH) by modulating cerebral oxidative stress. This study was performed to assess the effect of ATX on the Nrf2-ARE pathway and to explore the underlying molecular mechanisms of antioxidant properties of ATX in EBI after SAH. A total of 96 male SD rats were randomly divided into four groups. Autologous blood was injected into the prechiasmatic cistern of the rat to induce an experimental SAH model. Rats in each group were sacrificed at 24 h after SAH. Expressions of Nrf2 and heme oxygenase-1 (HO-1) were measured by Western blot and immunohistochemistry analysis. The mRNA levels of HO-1, NAD (P) H: quinone oxidoreductase 1 (NQO-1), and glutathione S-transferase-α1 (GST-α1) were determined by real-time polymerase chain reaction (PCR). It was observed that administration of ATX post-SAH could up-regulate the cortical expression of these agents, mediated in the Nrf2-ARE pathway at both pretranscriptional and posttranscriptional levels. Meanwhile, oxidative damage was reduced. Furthermore, ATX treatment significantly attenuated brain edema, blood–brain barrier (BBB) disruption, cellular apoptosis, and neurological dysfunction in SAH models. This study demonstrated that ATX treatment alleviated EBI in SAH model, possibly through activating the Nrf2-ARE pathway by inducing antioxidant and detoxifying enzymes.

## 1. Introduction

Astaxanthin (ATX) is a carotenoid widely found in algae and aquatic animals, which has powerful antioxidant activity [1]. Previous studies have revealed that ATX, with its antioxidative property, is beneficial as a therapeutic agent for various diseases both *in vivo* and *in vitro* without any side effects or toxicity [2,3,4]. Our earlier research has demonstrated that ATX administration after subarachnoid hemorrhage (SAH) can up-regulate the cortical endogenous antioxidant agents and prevent oxidative damage in experimental SAH models, thus alleviating early brain injury (EBI) [5], which is considered the most common cause of disability and death in SAH patients [6]. To date, however, there are few studies concerning the underlying molecular mechanisms of ATX in the SAH model.

Nrf2 is a basic leucine zipper redox-sensitive transcription factor, which transfers from the cytoplasm to the nucleus and binds to the ARE to activate transcription of several antioxidant genes under conditions of oxidative or xenobiotic stress [7,8,9]. The Nrf2-ARE pathway has been proven to play a beneficial role in EBI after SAH, possibly through inducing antioxidant and detoxifying enzymes to reduce cerebral oxidative stress [10].

In the previous studies concerning ATX and the Nrf2-ARE pathway, effects of ATX on Nrf2-ARE pathway activation had been investigated *in vivo* and *in vitro* models [1,11]. Tripathi and Jena [1] provided the evidence that ATX up-regulated the levels of Nrf2 and phase-II enzymes to exert its protective effects against cyclophosphamide-induced oxidative stress in rat liver. Zhang *et al.* [11] found that ATX could up-regulate the expression of Nrf2 in leukemia K562 cells in time- and dose-dependent manners. Previous studies had evaluated the activation of the Nrf2-ARE pathway, always using ATX as a pretreatment. However, there is no study investigating the effect of ATX on the Nrf2-ARE pathway in SAH. Thus, the purpose of this study was to explore whether ATX treatment post SAH could activate the Nrf2-ARE pathway and ameliorate EBI in the SAH model.

## 2. Results

### 2.1. General Observation

There were no significant differences detected in body weight, temperature, or injected arterial blood gas data among the groups.

### 2.2. Neurological Scores

**Figure 1:**
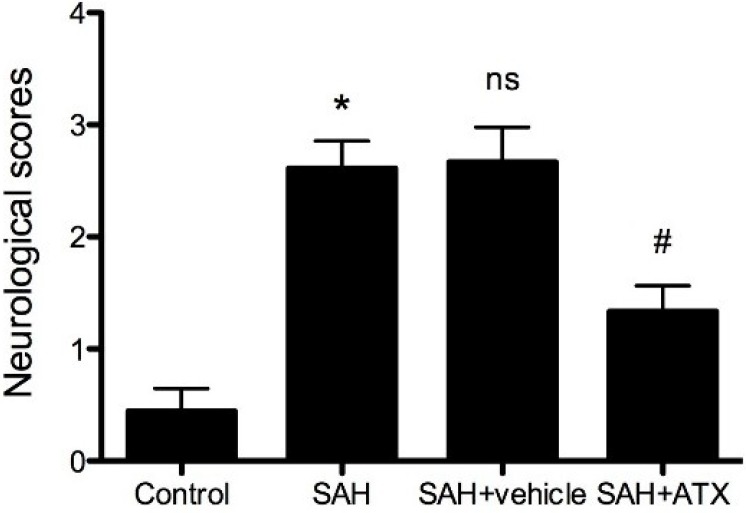
Neurological scores of each group. Data are expressed as means ± SEM. Neurologic impairments of rats due to SAH were improved because of the administration of Astaxanthin (ATX) after SAH. * *p* < 0.0001, compared with control group; **^#^** *p* < 0.05 compared with SAH group and SAH + vehicle group, respectively; **^ns^** *p* > 0.05, compared with SAH group.

An independent investigator blinded to the experimental groups and carried out a battery of tests looking at appetite, activity, and neurological deficits [12] before the rats were sacrificed. The deficits were scored with the scoring system shown in Table 1. Compared to the control group, neurological impairment caused by SAH was remarkable in the SAH group (Figure 1, *p* < 0.001). The SAH + ATX group showed better performance than either the SAH group or the SAH + vehicle group at 24 h after SAH, and the difference was statistically significant (Figure 1, *p* < 0.05). There were no significant differences between the SAH group and SAH + vehicle group (Table 1, *p* > 0.05).

**Table 1:** Behavior and activity scores.

Category	Behavior	Score
**Appetite**	Finished meal	0
	Left meal unfinished	1
	Scarcely ate	2
**Activity**	Walked and reached at least three corners of the cage	0
	Walked with some stimulation	1
	Almost always lying down	2
**Deficits**	No deficits	0
	Unstable walk	1
	Impossible to walk	2

### 2.3. Immunohistochemistry for Nrf2 and HO-1 Expression

**Figure 2:**
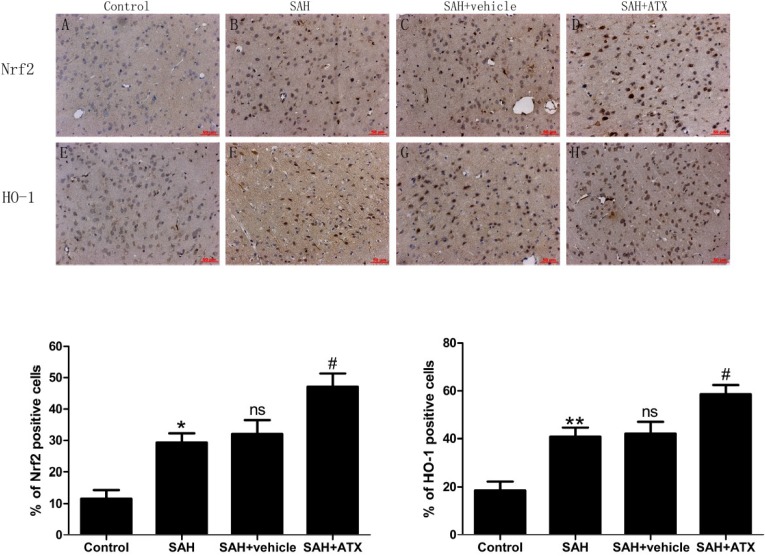
Representative photomicrographs showing Nrf2 (**A**–**D**) and HO-1 (**E**–**H**) immunohistochemistry staining of the temporal lobe in the experimental groups. As shown, a few Nrf2 and HO-1 stained cells were observed in the control group (*n* = 6), whereas numerous Nrf2 and HO-1 stained cells could be seen in the SAH group (*n* = 6) and SAH + vehicle group (*n* = 6). After ATX administration (*n* = 6), the immune-positive cells of Nrf2 and HO-1 in the cortex further increased following SAH. (**I**) Quantification analysis of immune reactive cells for Nrf2 and HO-1. The administration of ATX could induce Nrf2 and Nrf2-ARE pathway-related agent activation. Data are expressed as means ± SEM. * *p* < 0.05 compared with control group; ** *p* < 0.01 *vs.* control group; **^#^** *p* < 0.05 *vs.* SAH group and SAH + vehicle group, respectively; **^ns^** *p* > 0.05 *vs.* SAH group.

As is shown in Figure 2, a few Nrf2 and HO-1 positive cells stained brown and could be observed in the cortex of the control group. More Nrf2 and HO-1 positively immunostained cells were found in the SAH group and the SAH + vehicle group. In the SAH + ATX group, the number of Nrf2 and HO-1 positive cells was further increased compared with the SAH group or SAH + vehicle group.

### 2.4. Western Blot Analysis for Nrf2 and HO-1 Expression

**Figure 3:**
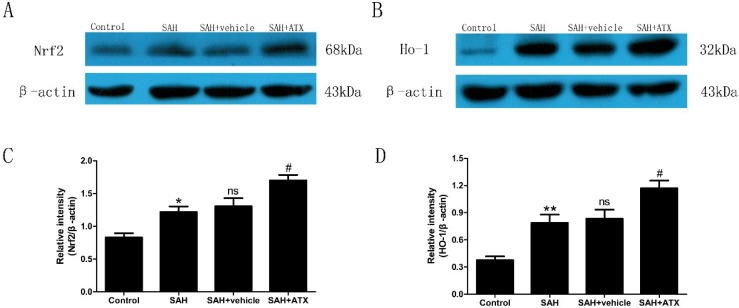
Representative autoradiogram of the Nrf2 (**A**) and HO-1 (**B**) in the experimental groups, and quantitative analysis of the Western blot results for Nrf2 (**C**) and HO-1 (**D**). SAH could induce a significant increase of Nrf2 and HO-1 expression in the rat cortex samples compared with the control group. After ATX administration, the protein levels of Nrf2 and HO-1 were further up-regulated. Data are expressed as means ± SEM. * *p* < 0.05 compared with control group; ** *p* < 0.01 *vs.* control group; **^#^** *p* < 0.05 *vs.* SAH and SAH + vehicle group, respectively; **^ns^** *p* > 0.05 *vs.* SAH group.

As is shown in Figure 3, low levels of Nrf2 and HO-1 were expressed in the control group. The levels of Nrf2 and HO-1 were significantly increased in the SAH and SAH + vehicle groups compared with the control group (*p* < 0.05 and *p* < 0.01, respectively) at 24 h after SAH. There was no significant difference between the SAH and the SAH + vehicle groups (*p* > 0.05) (Figure 3). After ATX treatment, the increased expression of Nrf2 and HO-1 was remarkable compared with that in the SAH group (*p* < 0.05) (Figure 3).

### 2.5. Quantitative Real-Time Polymerase Chain Reaction (PCR)

**Figure 4:**
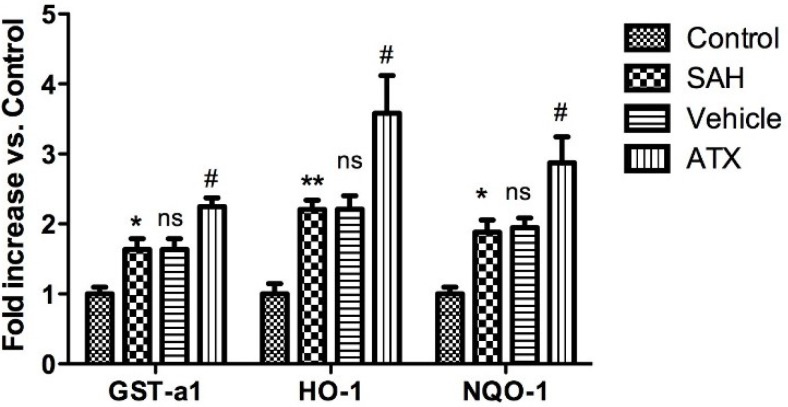
The cortical mRNA expressions of HO-1, NQO1, and GST-α1 in the control group (*n* = 6), SAH group (*n* = 6), SAH + vehicle group (*n* = 6), and SAH + ATX group (*n* = 6). SAH could induce a marked increase in HO-1, NQO1, and GST-α1 mRNA expressions in the rat cortex compared with the control group. After ATX administration, the mRNA expressions of Nrf2-ARE pathway-related agents were significantly up-regulated. * *p* < 0.05 *vs.* control group; ** *p* < 0.01 *vs.* control group; **^#^** *p* < 0.05 *vs.* SAH group and SAH + vehicle group, respectively; **^ns^** *P* > 0.05 *vs.* SAH group.

The cortical mRNA levels of HO-1, NQO1, and GST-α1 were significantly increased in the SAH and SAH + vehicle groups as compared with the control group (*p* < 0.01, *p* < 0.05 and *p* < 0.05, respectively). There are no significant differences in mRNA expressions of HO-1, NQO1, and GST-α1 between the SAH and SAH + vehicle groups (*p* > 0.05). The mRNA expressions of HO-1, NQO1, and GST-α1 in the cortex in the SAH + ATX group were markedly higher up-regulated than those in the SAH or SAH + vehicle group (*p* < 0.05) (Figure 4).

### 2.6. Enzyme Activity Assay for NQO1 and GST-α1

**Figure 5:**
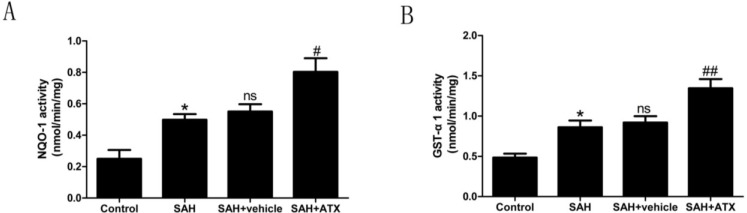
NQO1 (**A**) and GST-α1 (**B**) enzyme activities in the cortex in the control group (*n* = 6), the SAH group (*n* = 6), the SAH + vehicle group (*n* = 6), and the SAH + ATX group (*n* = 6). SAH could significantly elevate the activities of NQO1 and GST-α1. In the SAH + ATX group, the activities of NQO1 and GST-α1 were significantly up-regulated compared with the SAH + vehicle group. * *p* < 0.05 *vs.* control group; **^#^** *p* < 0.05 and **^##^** *p* < 0.01 *vs.* SAH + vehicle group; **^ns^** *p* > 0.05 compared with the SAH group.

Compared with the control group, enzyme activities of NQO1 and GST-α1 were up-regulated after SAH (*p* < 0.05) (Figure 5). On the other hand, ATX administration after SAH significantly increased NQO1 and GST-α1 activity in rat cortex. There was a significant difference between the SAH + ATX and the other groups (Figure 5).

**Figure 6:**
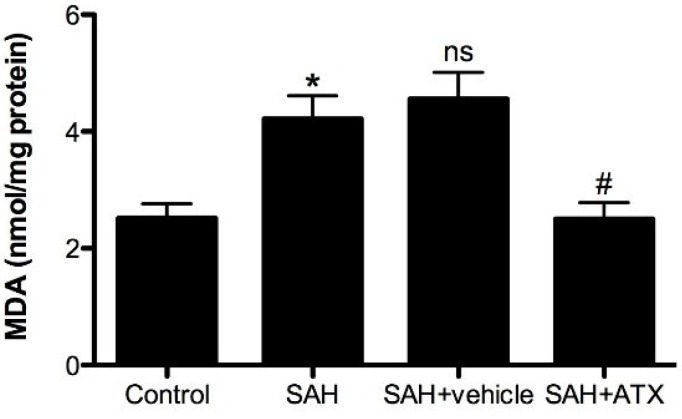
Malondialdehyde (MDA) levels in the control group (*n* = 6), SAH group (*n* = 6), SAH + vehicle group (*n* = 6), and SAH + ATX group (*n* = 6). As shown, the level of MDA in the cortex increased remarkably at 24 h after SAH. ATX treatment significantly suppressed the production of MDA after SAH. The values are expressed as mean ± SEM. * *p* < 0.05 compared with control group; ^#^ *p* < 0.05 compared with SAH group and SAH + vehicle group, respectively. **^ns^** *p* > 0.05 *vs.* SAH group.

### 2.7. Malondialdehyde (MDA) Levels

Malondialdehyde (MDA) is the end product of lipid peroxidation of polyunsaturated fatty acids in cellular membranes. MDA has been identified as a reliable marker of oxidative stress, which can represent cell damages indirectly. As shown in Figure 6, a significant increase in MDA levels was detected in the cortex samples at 24 h after SAH as opposed to the control group (*p* < 0.05). The level of MDA was significantly decreased with ATX treatment compared with the SAH and SAH + vehicle groups (*p* < 0.05, respectively).

### 2.8. Brain Water Content

**Figure 7:**
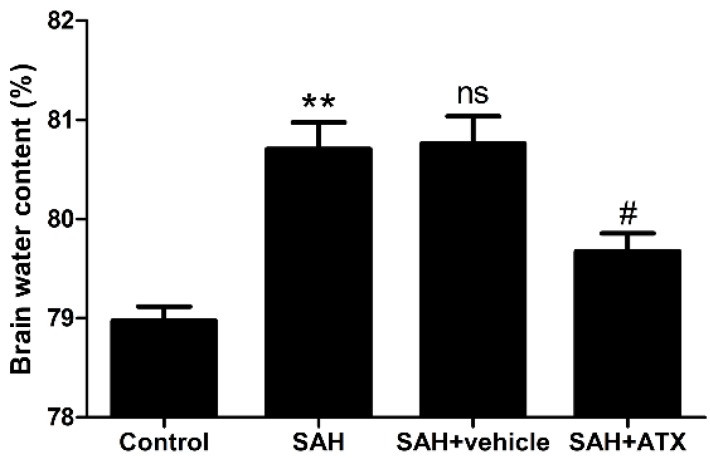
Percentages of brain water content in the control group (*n* = 6), SAH group (*n* = 6), SAH + vehicle group (*n* = 6), and SAH + ATX group (*n* = 6). As shown, the brain water content increased remarkably at 24 h after SAH. ATX treatment reduced brain water content significantly compared with the SAH group or SAH + vehicle group. The values are expressed as mean ± SEM. ** *p* < 0.01 compared with control group; **^#^** *p* < 0.05 compared with SAH group and SAH + vehicle group, respectively. **^ns^** *p* > 0.05 *vs.* SAH group.

Brain edema is a major cause of poor outcome after SAH. Blood-brain barrier (BBB) disruption was considered to be one of the most important pathogeneses resulting in brain edema [13]. In the present study, brain water content increased significantly at 24 h after SAH as compared with the control group (*p* < 0.01) (Figure 7). It was observed that ATX treatment significantly reduced brain edema as compared with the SAH and SAH + vehicle groups (*p* < 0.05, respectively) (Figure 7).

**Figure 8:**
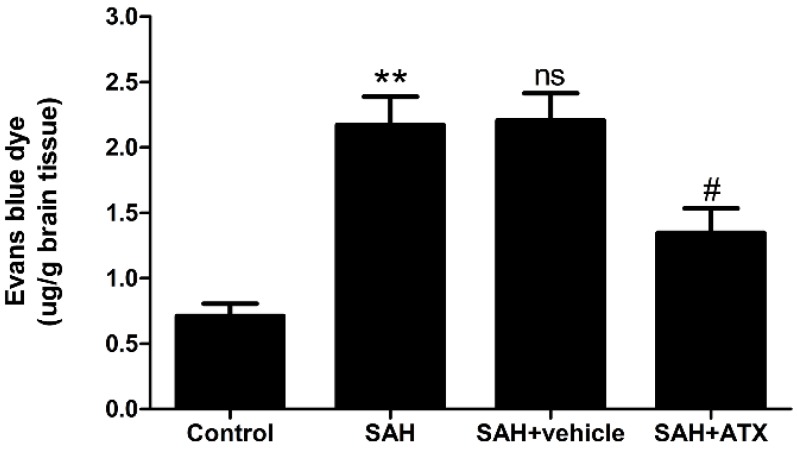
Evans blue (EB) extravasations in control the group (*n* = 6), SAH group (*n* = 6), SAH + vehicle group (*n* = 6), and SAH + ATX group (*n* = 6). SAH induced a significant increase in blood-brain barrier extravasation in the cortex compared with the control group, and ATX administration significantly decreased EB extravasation in this group compared with the SAH group or SAH + vehicle group. The values are expressed as mean ± SEM. ** *p* < 0.01 compared with control group; **^#^** *p* < 0.05 compared with SAH group and SAH + vehicle group, respectively; **^ns^** *p* > 0.05 *vs.* SAH group.

### 2.9. Blood-Brain Barrier Permeability

Evans blue (EB) extravasation as an indicator of BBB permeability was measured at 24 h after SAH, which is illustrated in Figure 8. The SAH group and SAH + vehicle group showed a significant increase of EB extravasation when compared with the control group (*p* < 0.01). As shown in Figure 8, ATX treatment significantly inhibited EB extravasation (*p* < 0.05), attenuating BBB permeability after SAH.

### 2.10. Terminal Deoxynucleotidyl Transferase-Mediate dUTP Nick end Labeling Staining (TUNEL) Assay

**Figure 9:**
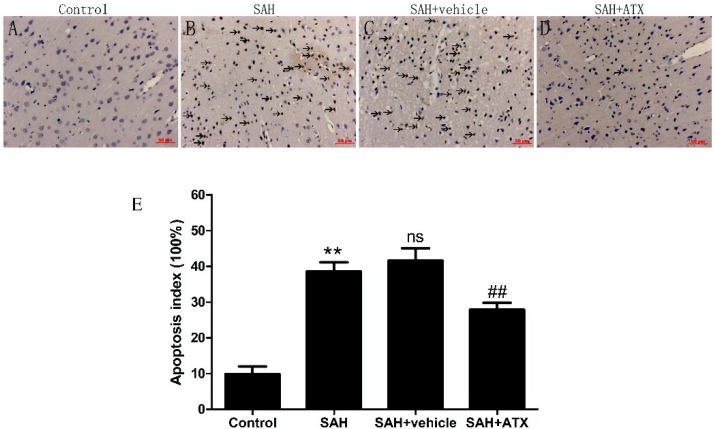
Representative photomicrographs of terminal deoxynucleotidyl transferase-mediate dUTP nick end labeling (TUNEL) staining (scar bar, 50 μm) and the apoptosis index of the cortex in the control group (*n* = 6), SAH group (*n* = 6), SAH + vehicle group (*n* = 6), and SAH + ATX group (*n* = 6). Control group (**A**) showing few apoptotic cells in the cortex. More TUNEL staining cells with the nucleus stained brown (arrows) could be observed in the SAH and SAH + vehicle groups (**B**,**C**). The proportion of apoptotic cells decreased significantly in the ATX group (**D**). (**E**) Apoptosis index. Bars represent the mean ± SEM. ** *p* < 0.01 *vs.* control group, **^##^** *p* < 0.01 *vs.* SAH + vehicle group, **^ns^** *p* > 0.05 *vs.* SAH group.

As illustrated in Figure 9, a few terminal deoxynucleotidyl transferase-mediate dUTP nick end labeling (TUNEL)-positive cells were found in the control group (Figure 9A). Compared with the control group, the percentages of apoptosis cells in the cortex significantly increased in the SAH and SAH + vehicle groups (*p* < 0.01) (Figure 9B,C). In the SAH + ATX group, the apoptosis index significantly decreased (*p* < 0.01) (Figure 9D) when compared with SAH and SAH + vehicle groups.

## 3. Discussion

The present study demonstrated that ATX activated the Nrf2-ARE pathway to up-regulate the expression of Nrf2-regulated enzymes against oxidative stress, such as HO-1, NQO1 and GST-α1, at 24 h after SAH. Accordingly, oxidative damage in the cortex induced by SAH was reduced. In the meantime, ATX administration attenuated brain edema, BBB disruption, cellular apoptosis, and neurological dysfunction in SAH models.

EBI is an important cause of high morbidity and mortality in patients suffering from SAH [14]. The exact mechanism of EBI remains obscure, although it is probably due to the elevation of intracranial pressure, cerebral perfusion disruption, brain edema, BBB breakdown, and neuronal cell death [12]. Accumulating evidence has demonstrated that oxidative stress plays an important role in the pathogenesis of EBI [15]. It has been reported that excessive reactive oxygen species (ROS) and reactive nitrogen species (RNS) including hydroxyl radical, superoxide anion, hydrogen peroxide, nitric oxide, and peroxynitrite are generated in the early period after SAH, consuming enzymatic and non-enzymatic antioxidant defense systems [16]. Moreover, these free radicals will promote lipid peroxidation, protein breakdown, and DNA damage, resulting in neuronal damage, cellular apoptosis, endothelial injury, and BBB disruption [15]. It may be a novel therapeutic target of SAH. 

ATX is a carotenoid containing an additional carboxyl group on each ring structure at the two extremities. It is a more effective antioxidant than vitamin E, by 100–1000 times. [17]. In addition, it has been reported that ATX possesses a variety of pharmacological properties without any side effects or toxicity, including anti-inflammatory, immunomodulatory, and anti-tumor activities [4,18,19]. Previous studies have proven that ATX plays a protective role in models of cardiovascular diseases including myocardial ischemia and reperfusion [20,21]. In a recent study, Shen *et al.* [2] demonstrated that ATX could reduce ischemia-related injury in brain tissue through inhibition of oxidative stress, reduction of glutamate release, and anti-apoptosis. Nevertheless, limited research has investigated the protective effect of ATX in SAH. Our previous study has proven that ATX treatment significantly attenuates brain edema and BBB disruption, reduces cellular apoptosis, and improves neurologic function at 24 h after SAH [5]. However, further studies should be performed to explore the underlying signaling pathway between ATX and SAH.

Increasing data indicate that Nrf2 plays a key role in antioxidant protection, which was revealed in various central nervous system diseases, such as cerebral ischemia, traumatic brain injury, and SAH [8,10,22,23]. Under these pathological conditions, Nrf2 translocates into the nucleus and then binds to ARE, transactivating a group of cytoprotective enzymes to protect cells against oxidative and xenobiotic damage. The protective effects of ATX on the Nrf2-ARE pathway activation in certain diseases have been demonstrated in previous studies [1,11,24]. Therefore, we hypothesized that ATX could also activate the Nrf2-ARE pathway in brain after SAH to alleviate EBI through inhibition of oxidative stress secondary to the up-regulation of antioxidant enzymes. In the present study, we found that SAH induced a significant increase in Nrf2 protein levels in the cortex. The mRNA levels of Nrf2-regulated gene products, HO-1, NQO1, and GST-α1, were also up-regulated at 24 h after SAH. This suggested triggering of the Nrf2-ARE pathway in the brain after SAH to exert antioxidant and detoxifying properties. Compared with SAH and SAH + vehicle groups, ATX administration significantly increased the Nrf2 protein level, up-regulated the expression of HO-1, NQO1 and GST-α1 at both pretranscriptional and posttranscriptional levels. HO-1, NQO1, and GST-α1 are potent antioxidant and detoxifying enzymes. HO-1 could reduce ROS production via generating biliverdin or bilirubin. NQO1 is able to protect cells against the adverse effects of quinones and related compounds. Unlike these two enzymes, GST-α1 performs the protective effect by up-regulating the content of endogenous glutathione [25,26]. Accordingly, ATX administration reduced the oxidant damage and ameliorated the EBI in the SAH models in this study. However, the mechanism of activation of the Nrf2-ARE pathway by ATX has not been completely elucidated. Several kinases, such as phosphoinositol-3 kinase (PI3K) and extra cellular signal-regulated protein kinase (ERK) have been reported to activate Nrf2 in response to some stimuli [27,28]. On the other hand, it has been reported that ATX induces activation of PI3K and ERK under some conditions [29,30]. It may be part of the mechanisms involved in the activation of the Nrf2-ARE pathway by ATX after SAH, which needs to be investigated in further studies.

In this study, we used the prechiasmatic cistern SAH model that is appropriate for EBI research since it produces similar pathological and pathophysiological processes to that in human with an acceptable mortality rate [31,32]. However, our study has several limitations. Firstly, ATX treatment was conducted only once and we do not know whether multiple treatments with different time courses would be effective. In addition, ATX may have other protective effects against EBI after SAH that were not evaluated in this study, such as anti-inflammatory and immunomodulatory properties. Lastly, Nrf2 knockout mice were not used in this research, although the improved outcome was regarded partly by beneficial effects of ATX through activating the Nrf2-ARE pathway. Therefore, comprehensive studies are still warranted.

## 4. Experimental Section

### 4.1. Animals

Male Sprague-Dawley rats (250–300 g) were purchased from the Animal Center of Jinling Hospital, Nanjing, China. They were acclimated in a humidified room and maintained on a standard pellet diet at the Animal Center of the Jinling Hospital for 10 days before the experiment. The temperature was maintained at about 25 °C in both the breeding room and the operation room. All procedures were approved by the Animal Care and Use Committee of Nanjing University and conformed to the Guide for the Care and Use of Laboratory Animals by the National Institutes of Health.

### 4.2. Experimental Design

**Figure 10:**
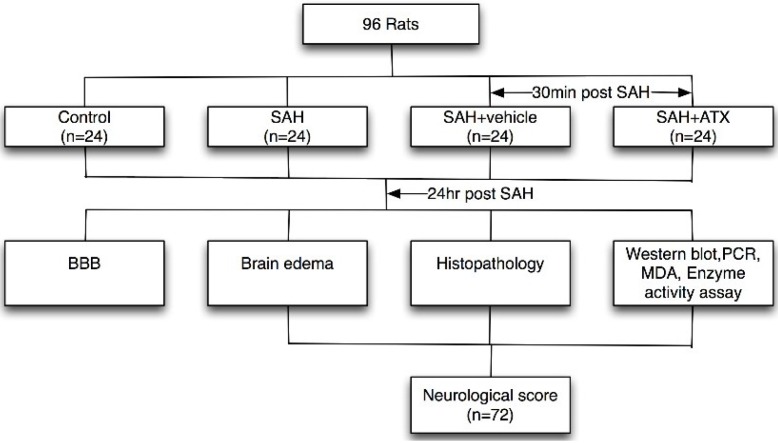
Schematic illustration of experimental design.

Ninety-six male SD rats were randomly divided into four groups: SAH group (*n* = 24); SAH + ATX group (*n* = 24); SAH + vehicle group (*n* = 24); and control group (*n* = 24). In the SAH + ATX group, ATX (20 μL of 0.1 mM dissolved in vehicle) was administrated at 30 min after SAH was induced. Rats of the SAH + vehicle group received equal volumes of vehicles (20 μL of 10% dimethylsulfoxide) at the corresponding time points. ATX or vehicle was administrated into the left ventricle (0.8 mm posterior, 1.5 mm lateral to the bregma, and 3.7 mm below dural) with a 25-μL Hamilton syringe (Shanghai Gaoge Industry & Trade Co. Ltd., Shanghai, China). The dose of ATX was chosen according to our previous study since we observed beneficial effects on reducing oxidant damage in the SAH models [5]. Rats in each group were killed at 24 h after SAH (Figure 10).

### 4.3. Prechiasmatic Cistern SAH Model

**Figure 11:**
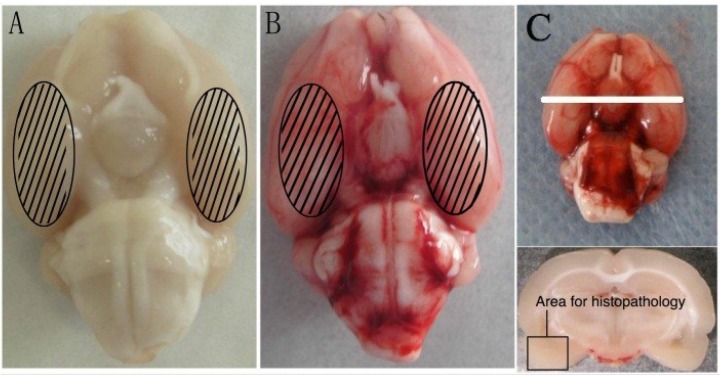
Representative photographs of brains taken for detection. (**A**) Rat brain of the control group and (**B**) the subarachnoid hemorrhage (SAH) group acutely removed following saline perfusion; (**A**–**C**) The detected areas of the cortex sample for relative assays are schematized.

The prechiasmatic cistern SAH model was produced according to a previous study under aseptic conditions [10]. The animal head was fixed in the stereotactic frame after the intraperitoneal anesthesia with pentobarbital sodium (50 mg/kg). A needle with a rounded tip and a side hole was tilted 45° in the sagittal plane and placed 7.5 mm anterior to bregma in the midline, with the hole facing the right side. It was advanced until the tip reached the skull base, about 2–3 mm anterior to the chiasma (10–12 mm away from the brain surface), and then retrieved 0.5 mm. Plugging the burr hole with bone wax prevented loss of cerebrospinal fluid and bleeding from the midline vessels. 0.25 ml non-heparinized fresh autologous arterial blood from the femoral artery was slowly injected into the prechiasmatic cistern for 20 s with a syringe pump. Animals in the control group were injected with 0.25 mL saline. The rats were sent back to their cages individually and received *ad libitum* access to food and water. Heart rate and rectal temperature were monitored, and the rectal temperature was kept at 37 °C ± 0.5 °C, using a warm pad if required. It was observed in the SAH models that the inferior basal temporal lobe was stained by blood (Figure 11).

### 4.4. Immunohistochemistry

Six rats in each group were sacrificed for immunohistochemistry and terminal deoxynucleotidyl transferase-mediate dUTP nick end labeling (TUNEL) staining, using the fixation perfusion method as described [7,33]. The whole brain was removed and immersed in the fixative solution after perfusion-fixation. The formalin-fixed tissues were embedded in paraffin and cut to thickness sections (4 μm) with a microtome. Immunohistochemistry was performed to determine the immunoreactivity of Nrf2 and HO-1. Briefly, sections were incubated at 4 °C overnight with primary antibody anti-Nrf2 (1:100; Abcam, Cambridge, MA, USA) and anti-HO-1 (1:200; Abcam, Cambridge, MA), then incubated with goat anti-rabbit horseradish peroxidase (HRP)-conjugated IgG (1:500, Santa Cruz Biotechnology, Santa Cruz, CA, USA) as secondary antibody. Sections were placed in an avidin-biotin-peroxidase complex enzyme after incubating. As following, 3,3′-diaminobenzidine (DAB) and hydrogen peroxide were used for staining.

### 4.5. TUNEL Assay

*In situ* cell death detection Kit POD (ISCDD, Boehringer Mannheim, Mannheim, Germany) was used for the TUNEL assay. The assay was performed according to the manufacturer’s instructions and previous studies. Sections were deparaffinized, hydrated, and washed with distilled water. The tissues were digested with 20 g/mL proteinase K (Boehringer Mannheim, Mannheim, Germany) at room temperature for 15 min. Endogenous peroxidase activity was blocked by incubation in 0.3% hydrogen peroxide/methanol in phosphate buffered saline (PBS) at 37 °C for 30 min. The sections were incubated with terminal deoxynucleotidyl transferase at 37 °C for 60 min to add the dioxigenin-conjugated dUTP to the 3′-OH ends of fragmented DNA. Anti-digoxigenin antibody peroxidase was applied to the sections to detect the labeled nucleotides. After that, the sections were stained with DAB and counterstained slightly with hematoxylin.

### 4.6. Cell Counting

One slice of every six serial cuttings was selected, and altogether six slices were collected and observed in the light microscope by two independent investigators blinded to the experimental groups. The number of positive cells in each section was counted in 10 microscope fields (at ×200 magnification) throughout the identical regions of the studied brain, and the mean per visual field was calculated.

### 4.7. Western Blot Analysis

The brain sample of rat was removed rapidly after saline perfusion and rinsed in 0.9% normal saline (4 °C) to wash away the blood and blood clot. And then the tissue was frozen in liquid nitrogen for molecular biological and biochemical experiments. The protein concentration was estimated by the Bradford method with the Nanjing Jiancheng protein assay kit (Nanjing Jiancheng Bioengineering Institute, Nanjing, China). The frozen sample was homogenized in 20 mM Tris (pH 7.6) which contains 0.2% SDS, 1% Triton X-100, 1% deoxycholate, 1 mM phenylmethylsulphonyl fluoride (PMSF), and 0.11 IU/mL aprotinin (all from Sigma-Aldrich, Inc., St. Luis, MO, USA) for HO-1 analysis. Lysate was centrifuged for 20 min (at 12,000× *g*, 4 °C), and the supernatant was collected. Nuclear protein was extracted for Nrf2 analysis as described [33,34]. Equal amount of protein per lane was separated by 10% sodium dodecyl sulfate polyacrylamide gel electrophoresis (SDS-PAGE) and transferred to polyvinylidene-difluoride membrane (Bio-Rad Lab, Hercules, CA, USA). The membrane was blocked in 5% skimmed milk for 2 h at room temperature, and incubated overnight at 4 °C with primary antibodies against Nrf2 (1:1000, Abcam, Cambridge, MA, USA), HO-1 (1:2000, Abcam) and GAPDH (1:5000, Abcam) in PBS + Tween 20 (PBST) containing 1% skimmed milk. After the membrane was washed for 10 min each for four times in PBST, it was incubated with goat anti-rabbit HRP-conjugated IgG (diluted 1:1000 in phosphate-buffered saline/Tween (PBST), Abcam, Cambridge, UK) for two hours at room temperature. The blotted protein bands were visualized by enhanced chemiluminescence (ECL) Western blot detection reagents (Amersham, Arlington Heights, IL, USA) and were exposed to X-ray films. Developed films were digitized with an Epson Perfection 2480 scanner (Seiko Corp, Nagano, Japan). Optical densities were obtained using the UN-Scan-It 6.1 software (Silk Scientific Inc., Orem, UT, USA) and the data were normalized to β-actin.

### 4.8. Quantitative Real-Time PCR

Real-time PCR was used to analyze the mRNA levels of HO-1, NQO1 and GST-α1. Total RNA from brain sample was extracted using Biomiga Tissue RNA Kit (Biomiga Inc., San Diego, CA, USA) following the manufacturer’s instructions. Reverse transcription was performed using Promega (Madison, WI, USA) reagents. Real-time PCR analysis proceeded by real-time DNA analysis system (Genetimes Technology, Inc., Shanghai, China), using real-time SYBR Green PCR technology.

Forward and reverse primers were 5′-GCGAAACAAGCAGAACCCA-3′ and 5′-GCTCAGGATGAGTACCTCCCA-3′ for HO-1 (185 bp); 5′-GCGTCTGGAGACTGTCTGGG-3′ and 5′-CGGCTGGAATGGACTTGC-3′ for NQO1 (170 bp); 5′-CGGTACTTGCCTGCCTTTG-3′ and 5′-ATTTGTTTTGCATCCACGGG-3′ for GST-α1 (248 bp); 5′-CCCATCTATGAGGGTTACGC-3′ and 5′-TTTAATGTCACGCACGATTTC-3′ for β-actin (150 bp).

### 4.9. Enzyme Activity Assay

The frozen cortex tissue was homogenized in 10 mM Tris-HCl (pH 7.8) and centrifuged for 15 min (at 12000× *g*, 48 °C). The supernatant was collected, and NQO1 enzyme activity was measured by the reduction of the dicumarol-sensitive fraction of 2, 6-dichlorophenol-indo-phenol with spectrophotometry at 540 nm. GST-α1 enzyme activity was determined by conjugation reaction of 1-chloro-2, 4-dinitrobenzene and glutathione, calculating slope elevation of extinction coefficient at 340 nm.

### 4.10. MDA Level Determination

The MDA level was determined using the method described in a previous study [5,35]. The MDA concentrations were measured as nanomoles/milligram. The principle of the MDA level assay depends on the lipid peroxidation products with thiobarbituric acid and formation of products called thiobarbituric acid-reacting substances, which give maximum absorbance at the 535 nm.

### 4.11. Brain Water Content

Rats were anesthetized and decapitated at 24 h after SAH. Brains were rapidly removed and immediately weighed as wet weight. Samples were subsequently placed in an oven for 72 h at 100 °C and weighed again to get the dry weight. The brain water content (%) was calculated as a percentage using the following method: (wet weight − dry weight)/wet weight × 100% [36].

### 4.12. Blood-Brain Barrier Permeability

BBB permeability was assessed by EB extravasation at 24 h after SAH. EB dye (2%; 4 mL/kg) was injected over two minutes into the right femoral vein to circulate for 60 min. Rats were then anaesthetized and perfused from the left cardiac ventricle to the right cardiac atrium with normal saline to replace the EB dye. The brains were removed after the decapitations and then homogenized in phosphate-buffered saline. Trichloroacetic acid was added to deposit the protein. After that, samples were cooled and centrifuged. The supernatant was dislodged to measure absorbance of EB at 620 nm by a spectrophotometer (752S UV-Vis Spectrophotometer, Lengguang Tech., Shanghai, China).

### 4.13. Statistical Analysis

All data were presented as mean ± SEM. SPSS 20.0 was used for statistical analysis of the data. Kruskal-Wallis test followed by the Dunnis *post hoc* test were used to compare the differences among groups for neurological scores. The other data were subjected to one-way ANOVA and Tukey’s multiple comparison test. Differences with *p* < 0.05 were considered statistically significant.

## 5. Conclusions

In summary, to the best of our knowledge, this study was the first one to demonstrate the effects of ATX on the Nrf2-ARE pathway in the cerebral cortex after SAH. The results of the present study suggest that ATX treatment 30 min after SAH can result in further activation of the Nrf2-ARE pathway and, accordingly, ameliorate EBI after SAH. The benefit of ATX treatment might be caused by the up-regulation of Nrf2 trans-activating enzymes to suppress oxidative damage, which was one of its potential mechanisms. These findings are novel for the therapeutic strategy of EBI after SAH.
